# Supplementary material for: High-throughput Plasmodium falciparum hrp2 and hrp3 gene deletion typing by digital PCR to monitor malaria rapid diagnostic test efficacy
Source: eLife. 2022 Jun 28;11:e72083. doi: 10.7554/eLife.72083 (PMC9246365; doi:10.7554/eLife.72083)
Supplement: Supplementary file 1. [file elife-72083-supp1.docx]

**High-throughput *Plasmodium falciparum hrp2* and *hrp3* gene deletion typing by digital PCR to monitor malaria rapid diagnostic test efficacy**

Claudia A. Vera-Arias *et al.*

**Supplementary File 1**

**ddPCR assays**

Instrument: BioRad QX200 Droplet Digital PCR System

Supermix from BioRad

All primers and probes at 10 µM

***hrp2* exon 2 ddPCR**

|  | [uL] |  | Cycling conditions | |
| --- | --- | --- | --- | --- |
| SuperMix for Probes (no dUTP) | 11 |  |  |  |
| hrp2_Exon 2_fwd | 1.76 |  | 95° | 10 min |
| hrp2_Exon 2_rev | 1.76 |  | 94° | 30 sec |
| hrp2_Exon 2_Probe | 0.88 |  | 56° | 1 min => 44 x back to step 2 |
| tRNA_fwd | 0.44 |  | 98° | 10 min |
| tRNA_rev | 0.44 |  | 4° | hold |
| tRNA_Probe | 0.22 |  |  |  |
| H2O | 3.5 |  |  |  |
| DNA | 2 |  |  |  |

***hrp2* exon 1 ddPCR**

|  | [uL] |  | Cycling conditions | |
| --- | --- | --- | --- | --- |
| SuperMix for Probes (no dUTP) | 11 |  |  |  |
| hrp2-exon1_fwd | 1.76 |  | 95° | 10 min |
| hrp2-exon1_rev | 1.76 |  | 94° | 30 sec |
| hrp2-exon1_Probe | 0.88 |  | 54° | 1 min => 44 x back to step 2 |
| tRNA_fwd | 0.44 |  | 98° | 10 min |
| tRNA_rev | 0.44 |  | 4° | hold |
| tRNA_Probe | 0.22 |  |  |  |
| H2O |  |  |  |  |
| DNA | 2 |  |  |  |

***hrp3* ddPCR**

|  | [uL] |  | Cycling conditions | |
| --- | --- | --- | --- | --- |
| SuperMix for Probes (no dUTP) | 11 |  |  |  |
| hrp3_fwd | 0.66 |  | 95° | 10 min |
| hrp3_rev | 0.66 |  | 94° | 30 sec |
| hrp3_Probe | 0.44 |  | 55° | 1 min => 44 x back to step 2 |
| tRNA_fwd | 0.44 |  | 4° | hold |
| tRNA_rev | 0.44 |  |  |  |
| tRNA_Probe | 0.22 |  |  |  |
| H2O | 6.14 |  |  |  |
| DNA | 2 |  |  |  |

**Nested PCR assays**

Instrument: BioRad T100 Thermal Cycler

Polymerase, buffer, and dNTPs from Solis Biodyne

All primers at 10 µM

***hrp2* nested PCR**

| **Primary PCR** | [uL] |  | Cycling conditions | |
| --- | --- | --- | --- | --- |
| Taq FirePol (Solis Biodyne) | 0.15 |  |  |  |
| Buffer B (Solis Biodyne) | 1.5 |  | 94° | 10 min |
| MgCl2 (Solis Biodyne) | 1.5 |  | 94° | 50 sec |
| dNTPs (Solis Biodyne) | 1.3 |  | 55° | 50 sec => 39x back to step 2 |
| Primer hrp2_F1 | 0.2 |  | 70° | 1 min |
| Primer hrp2_R1 | 0.2 |  | 70° | 5 min |
| H2O | 8.25 |  |  |  |
| DNA | 2 |  |  |  |
|  |  |  |  |  |
| **Nested PCR** | **[uL]** |  | Cycling conditions | |
| Taq FirePol (Solis Biodyne) | 0.15 |  |  |  |
| Buffer B (Solis Biodyne) | 1.5 |  | 94° | 10 min |
| MgCl_2_ (Solis Biodyne) | 1.5 |  | 94° | 50 sec |
| dNTPs (Solis Biodyne) | 1.3 |  | 55° | 50 sec => 39x back to step 2 |
| Primer hrp2_F2 | 0.2 |  | 70° | 1 min |
| Primer hrp2_R1 | 0.2 |  | 70° | 5 min |
| H2O | 8.25 |  |  |  |
| DNA | 2 |  |  |  |

**Primer sequences**

Hrp2 F1 5′-CAAAAGGACTTAATTTAAATAAGAG-3′

Hrp2 R1 5′-AATAAATTTAATGGCGTAGGCA-3′

Hrp2 F2 5′-ATTATTACACGAAACTCAAGCAC-3′

***msp2* nested PCR**

Polymerase, buffer, and dNTPs from Solis Biodyne

All primers at 10 µM

| **Primary PCR** | [uL] |  | Cycling conditions | |
| --- | --- | --- | --- | --- |
| ddH2O | 7.85 |  |  |  |
| Buffer B (Solis Biodyne) | 1.5 |  | 95˚ | 1 min |
| MgCl2 25 mM (Solis Biodyne) | 1.5 |  | 95˚ | 30 sec |
| dNTPs 2.5 mM (Solis Biodyne) | 1.2 |  | 55˚ | 30 sec |
| Primer *msp2_*S2-fw | 0.4 |  | 72˚ | 1 min => 34 x back to step 2 |
| Primer *msp2*_S3-rev | 0.4 |  | 72˚ | 5 min |
| Taq FirePol (Solis Biodyne) | 0.15 |  |  |  |
| DNA | 2 |  |  |  |
|  |  |  |  |  |
| **Nested PCR** | [uL] |  | Cycling conditions | |
| ddH2O | 8.975 |  |  |  |
| Buffer B (Solis Biodyne) | 1.5 |  | 95˚ | 1 min |
| MgCl2 25 mM (Solis Biodyne) | 1.5 |  | 95˚ | 30 sec |
| dNTPs 2.5 mM (Solis Biodyne) | 1.2 |  | 55˚ | 30 sec |
| Primer M5_Fc27 (10 uM) | 0.15 |  | 72˚ | 1 min => 34 x back to step 2 |
| Primer N5 3D7 (10 uM) | 0.225 |  | 72˚ | 5 min |
| Primer S1-tail fw | 0.3 |  |  |  |
| Taq FirePol (Solis Biodyne) | 0.15 |  |  |  |
| Primary PCR as template | 1 |  |  |  |

**Primer sequences**

*msp2_*S2-fw 5’- GAAGGTAATTAAAACATTGTC-3’

*msp2*_S3-rev 5’- GAGGGATGTTGCTGCTCCACAG -3’

*msp2_*S1Tail-fw 5’- GCTTATAATATGAGTATAAGGAGAA -3’

*msp2_FC27 type_*M5-rev 5’- GCATTGCCAGAACTTGAA -3’

*msp2_3D7 type_*N5 -rev 5’- VIC-CTGAAGAGGTACTGGTAGA -3’
